# Bioinformatic Prediction of Possible Targets and Mechanisms of Action of the Green Tea Compound Epigallocatechin-3-Gallate Against Breast Cancer

**DOI:** 10.3389/fmolb.2017.00043

**Published:** 2017-06-30

**Authors:** Xinqiang Song, Mu Zhang, Lei Chen, Qingsong Lin

**Affiliations:** ^1^Department of Biological Sciences, Xinyang Normal UniversityHenan, China; ^2^Department of Biological Sciences, National University of SingaporeSingapore, Singapore; ^3^Hospital Attached to Xinyang Normal UniversityXinyang, China

**Keywords:** epigallocatechin-3-gallate, breast cancer, ingenuity pathway analysis, bioinformatics analysis, targets

## Abstract

Epigallocatechin-3-gallate (EGCG), a bioactive compound in green tea, is the most abundant and biologically active catechin, and it exerts multiple effects in humans through mechanisms that remain to be clarified. The present study used bioinformatics to identify possible mechanisms by which EGCG reduces risk of breast cancer. Possible human protein targets of EGCG were identified in the PubChem database, possible human gene targets were identified in the NCBI database, and then both sets of targets were analyzed using Ingenuity Pathway Analysis to predict molecular networks affected by EGCG in breast cancer. The results suggest that signaling proteins affected by EGCG in breast cancer, which include JUN, FADD, NFKB1, Bcl-2, GNAO1, and MMP14, are involved primarily in cell death and survival; DNA replication, recombination and repair; and the cell cycle. The main networks affected by EGCG are predicted to involve the cell cycle; cellular assembly and organization; DNA replication, recombination and repair; and cell death and survival. These results identify several specific proteins and pathways that may be affected by EGCG in breast cancer, and they illustrate the power of integrative bioinformatics and chemical fragment analysis for focusing mechanistic studies.

## Introduction

Breast cancer is frequent in women, and incidence continues to increase, even though improvements in prevention, mammography-based screening, and treatments (e.g., endocrine therapy) have reduced patient mortality. Approximately one in eight women in the US will develop invasive breast cancer during their lifetime, and breast cancer incidence among women in the UK has increased by 6% in recent years (Harbeck and Gnant, 2017). Incidence is even increasing in countries where it has been relatively low, such as Japan and China (Xiang et al., 2016).

In 1997, epidemiological work in Japan suggested that drinking green tea, which is prepared from *Camellia sinensis* leaves, could reduce risk of breast cancer among women, especially those drinking more than 10 cups of green tea per day (Imai et al., 1997). Since then, numerous cohort studies and case-control studies in China, USA, and Singapore have confirmed an association between green tea consumption and reduction of breast cancer risk (Wu et al., 2003; Yuan et al., 2005; Zhang et al., 2007, 2009; Ganmaa et al., 2008; Inoue et al., 2008; Kumar et al., 2009; Shrubsole et al., 2009; Chen et al., 2010; Dai et al., 2010; Li et al., 2016). For example, one study involving 5082 women in USA showed that women who drank at least three cups of green tea per day had 37% lower risk of breast cancer than women who did not drink any tea (Kumar et al., 2009).

The ability of green tea to protect against breast cancer appears to be mediated by catechins, which are polyphenols accounting for 30–40% of the dry weight of brewed green tea. The four major catechins in green tea are (–)-epigallocatechin-3-gallate (EGCG), (–)-epigallocatechin, (–)-epicatechin gallate, and (–)-epicatechin, and EGCG is both the most abundant and most biologically active (Du et al., 2012; Kanwar et al., 2012). EGCG and other compounds extracted from green tea have been shown to suppress carcinogen-induced production of reactive oxygen species (ROS) and DNA damage, as well as alter cell signaling pathways (Ruch et al., 1989; Kaur et al., 2007; Rathore et al., 2012). Some researchers (Jones and Takai, 2001; Fang et al., 2003; Lee et al., 2005) found that EGCG can suppress cancer by inhibiting DNA methylation, anti-proliferation and inducing cancer cell apoptosis. One or several of these mechanisms may help explain how green tea extracts reduce risk of breast cancer.

The present study explored possible downstream proteins and pathways that EGCG may affect, in an effort to guide more detailed mechanistic studies to elucidate how EGCG reduces risk of breast cancer. This study used integrative bioinformatics analysis to bring together predictions of protein and pathway targets, followed by Ingenuity Pathway Analysis to build these predicted targets into a network model of interacting molecules that may help explain the presumably complex effects that green tea exerts in breast cancer.

## Materials and methods

### Dataset of breast cancer-related genes

The National Center for Biotechnology Information (NCBI) Gene Database (http://www.ncbi.nlm.nih.gov/gene; up to 15 December, 2016), which integrates information from a wide variety of species, was searched for genes related to breast cancer using the search term “breast cancer.” Search hits were filtered to retain only *Homo sapiens* genes (Supplementary Table S1).

### Dataset of EGCG-targeted proteins

The PubChem database of small molecules (http://pubchem.ncbi.nlm.nih.gov; up to 15 December, 2016), including the Compound, Bioassay, and Substance sub-databases, was searched for proteins shown in bioassays to be affected by EGCG (CID:65064) or predicted to be affected by EGCG based on similarity with known binders. Search hits were limited to *Homo sapiens* proteins (Supplementary Table S2).

### Prediction of interaction networks affected by EGCG

A network of interacting molecules was built using on-line Ingenuity Pathway Analysis (IPA, www.ingenuity.com) based on the dataset of human genes related to breast cancer and the dataset of EGCG-targeted human proteins (“focus molecules”). Based on the functions of these focus molecules, Pathway Analysis generated a set of networks likely to be affected by EGCG. Molecules were represented as nodes with different shapes depending on their function; and lines were drawn between nodes shown to be biologically related in at least one reference from the literature, a textbook, or other canonical information stored in the Ingenuity Knowledge Base.

**Figure 1:**
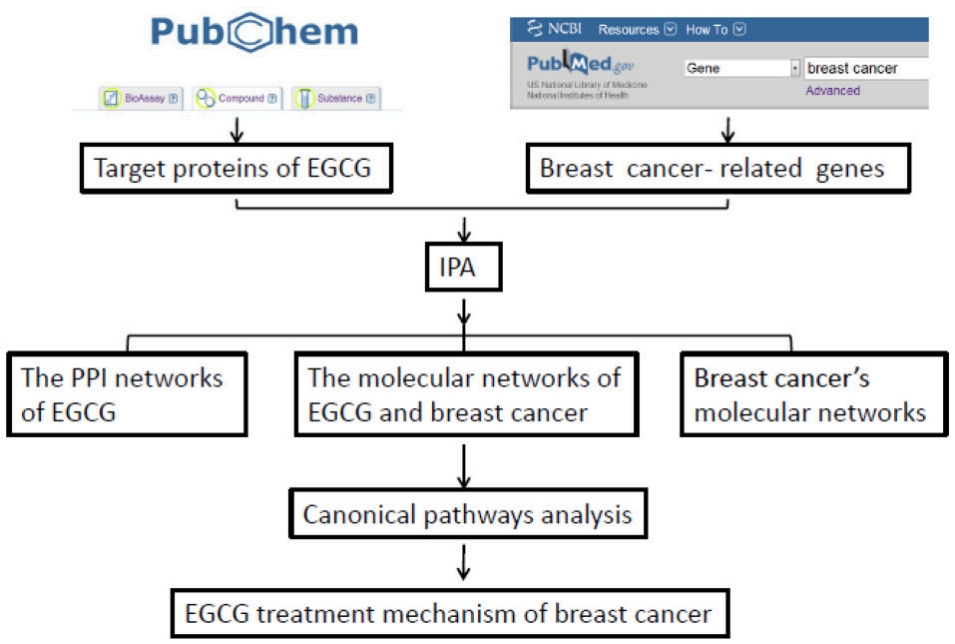
Flow diagram of network-based analysis of possible molecular mechanisms of EGCG in reducing risk of breast cancer. IPA, Ingenuity Pathway Analysis; PPI, protein-protein interactions.

The networks generated by IPA were scored according to the significance of the molecules in the network, then the “Compare” module within IPA was used to determine the significance of the association between focus molecules and canonical pathways, based on Fisher's exact test. Finally, we overlaid the two networks to discover the most likely targets of EGCG in breast cancer (Figure 1).

## Results

### Breast cancer-related gene networks and their functions

A total of 3,237 human genes linked to breast cancer were identified in the GenBank database (Supplementary Table S1), and the encoded proteins were assembled into a set of 25 networks using IPA. These pathways involve primarily cell death and survival, cellular growth and proliferation, inflammatory response, cell-to-cell signaling and interaction, metabolic disease, as well as cellular assembly and organization (Supplementary Table S3, Supplementary Figure S1).

### EGCG-targeted protein networks and their functions

A total of 65 human proteins targeted by EGCG were identified from the PubChem database (Supplementary Table S2) and their GenInfo Identifier numbers were imported into IPA, which generated protein-protein interaction networks (Supplementary Table S4, Supplementary Figure S2). Proteins targeted by EGCG participate primarily in the cell cycle; cellular assembly and organization; DNA replication, recombination and repair; cell death and survival; gastrointestinal disease; hepatic system disease; cell morphology; nervous system development and function; organ morphology; and carbohydrate metabolism.

### Network overlap to predict pathways affected by EGCG in breast cancer

**Figure 2:**
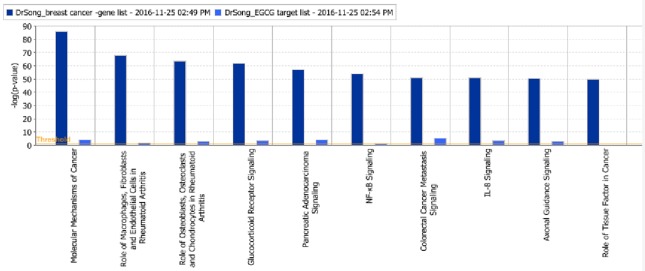
Overlap between signaling pathways linked to breast cancer (dark blue) and signaling pathways targeted by EGCG (light blue). Details are listed in Supplementary Figure S3.

The “Canonical Pathway” module of IPA identified 485 signaling pathways linked to breast cancer and 246 targeted by EGCG, with 235 signaling pathways shared between the two sets. These overlapping pathways primarily involve molecular mechanisms of cancer, inflammation, glucocorticoid receptor signaling, and cytokine signaling (Figure 2).

**Figure 3:**
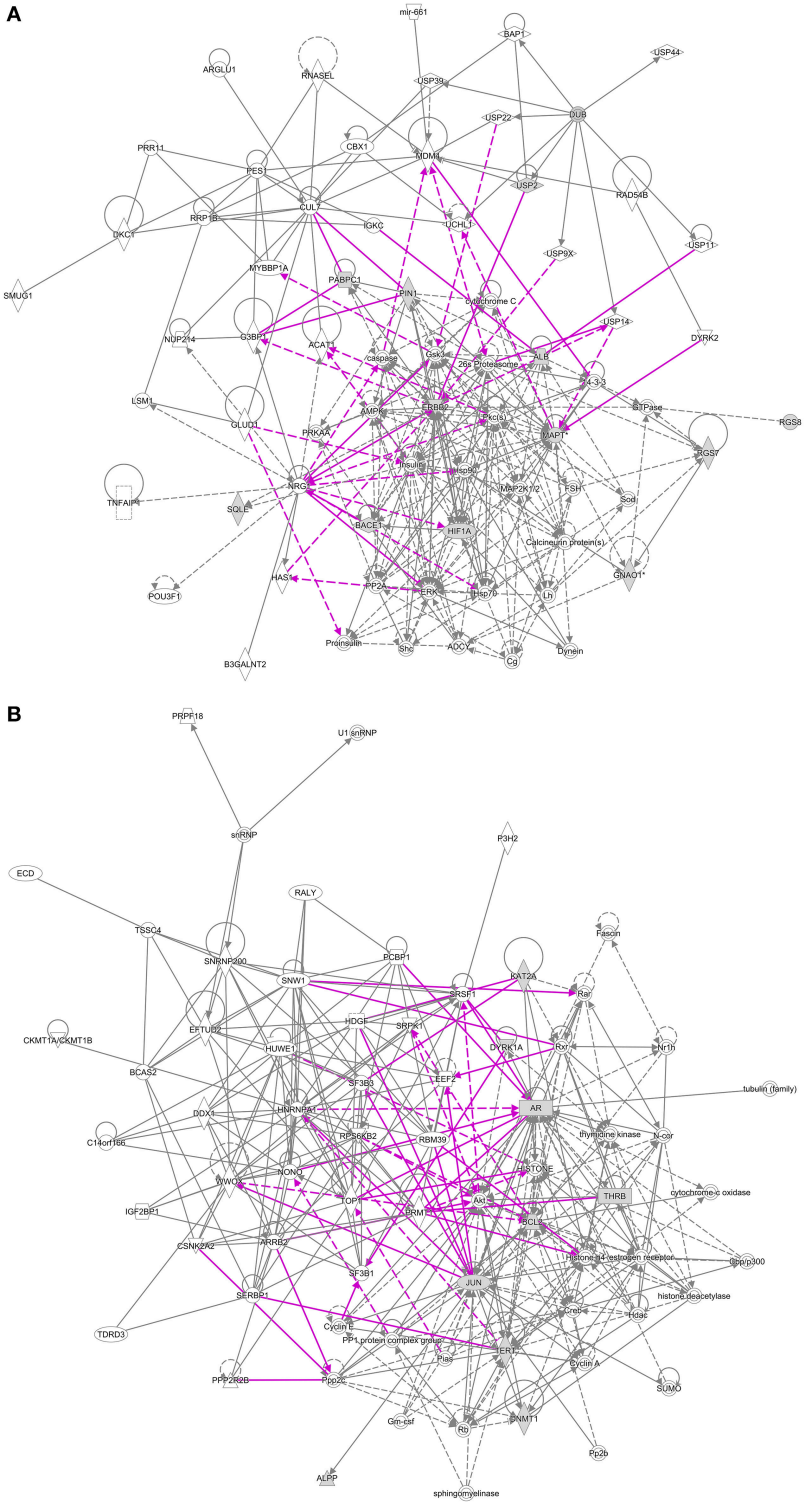
Overlap between networks linked to breast cancer and networks targeted by EGCG. Proteins linked to breast cancer are represented as black lines; EGCG-targeted proteins, as purple lines. **(A)** Shared networks involved in post-translational modification, protein synthesis, cell morphology, and nervous system development. **(B)** Shared networks involved in post-transcriptional modification, cell death and survival, cellular development, cell cycle, cancer, and endocrine disorders.

The “Networks” module of IPA identified 25 networks linked to breast cancer and 6 targeted by EGCG, with 5 networks shared between the two sets (Figure 3, Supplementary Table S5). These networks are primarily involved in cell death and survival; DNA replication, recombination and repair; cell cycle; cellular assembly and organization; post-translational modifications related to development; post-transcriptional modifications; and protein synthesis.

### Prediction of specific EGCG target proteins in breast cancer

**Figure 4:**
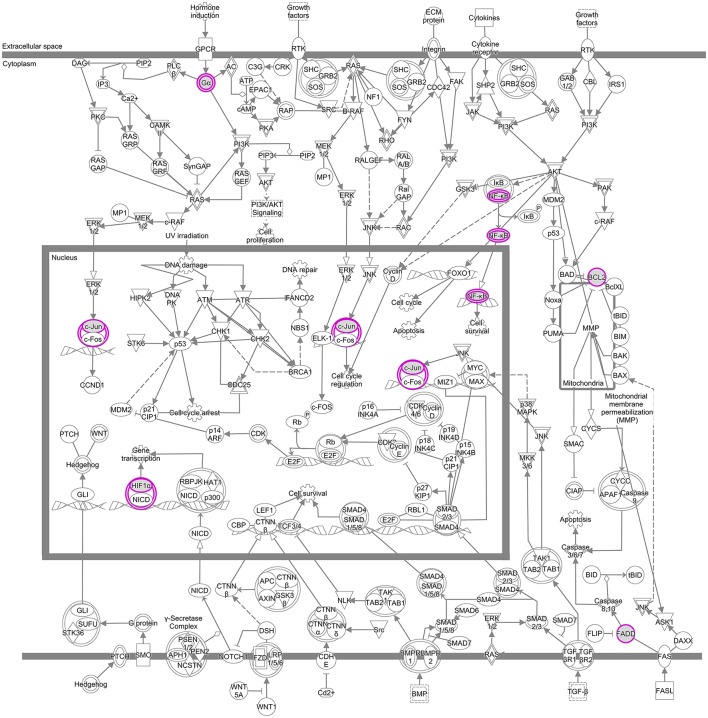
Signaling pathways assigned to the IPA category “molecular mechanisms of cancer” that have been linked to breast cancer and targeted by EGCG. Proteins directly targeted by EGCG are represented as purple boxes.

Proteins linked to breast cancer and targeted by EGCG participate in several canonical pathways underlying a range of biological activities. To demonstrate the ability of this integrative bioinformatics approach to propose specific protein targets for detailed mechanistic studies, we selected one pathway in the IPA category “molecular mechanisms of cancer” that was linked to breast cancer and targeted by EGCG. Several nodes in this pathway emerged as potential direct targets of EGCG in breast cancer: JUN, FADD, NFKB1, Bcl-2, GNAO1, and MMP14 (Figure 4, Supplementary Table S6). These potential target molecules also appeared in other canonical pathways linked to breast cancer and targeted by EGCG (Supplementary Figures S4, S5).

## Discussion

Here we applied an integrative bioinformatics approach drawing on free, publicly available databases of genes linked to breast cancer and of proteins or protein fragments known or predicted to be affected by EGCG. Drawing on the large size of both databases, we were able to identify numerous signaling pathways and networks potentially linked to breast cancer and potentially regulated by EGCG. Then we were able to predict several specific proteins likely to be affected by EGCG in breast cancer. These are strong leads for detailed mechanistic studies, illustrating the power of this bioinformatics-based “panning” or “screening” to guide studies of how EGCG may help reduce risk of breast cancer.

Our network analysis implicated several pathways by which EGCG may reduce breast cancer risk, involving cell death and survival; DNA replication, recombination and repair; cell cycle; cellular assembly and organization. These results are consistent with several studies *in vitro* and *in vivo* suggesting that EGCG exerts anti-carcinogenic activity by protecting DNA from ROS-induced damage and generally alleviating ROS stress (Ruch et al., 1989; Kaur et al., 2007), as well as by inhibiting DNA cleavage (Rathore et al., 2012). Our results are also consistent with work suggesting that EGCG suppresses proliferation and induces apoptosis by down-regulating anti-apoptotic factors such as B cell lymphoma 2 (Bcl-2), Bcl-xL, and vimentin (Leone et al., 2003; Ermakova et al., 2005), as well as by inhibiting NF-κB, JAK/STAT, and PI3K pathways (Surh et al., 2001; Lambert et al., 2010; Van Aller et al., 2011; Senggunprai et al., 2014).

Our analysis of canonical pathways in IPA suggests that EGCG may reduce breast cancer risk by altering pathways involved in molecular mechanisms of cancer, inflammatory signaling, glucocorticoid receptor signaling, and cytokine signaling. In particular, we identified JUN, FADD, NFKB1, Bcl-2, GNAO1, and MMP14 as potential targets of EGCG in breast cancer.

It was reported that p21-activated protein kinase 1 induced the invasion of gastric cancer cells through c-Jun NH2-terminal kinase-mediated activation of matrix metalloproteinase-2, and FADD protected pancreatic cancer cells from drug-induced apoptosis (Li et al., 2017; Zhang et al., 2017).

Researchers Pei et al. (2013) and Stawowczyk et al. (2017) had shown that down-regulation of GNAO1 increased cell proliferation and MMP14 promoted lung cancer by cleavage of heparin-binding EGF-like growth factor. How EGCG may work against these proteins needs more experiments to verify it. In agreement with our results, EGCG has been shown to bind with high affinity to Bcl-2, as well as to down-regulate Bcl-2 and NF-κB. The remaining targets that we identified have not previously been reported in the literature, to the best of our knowledge. This means that they may be novel potential targets that merit validation and, if positive, detailed mechanistic studies.

## Conclusion

Integrative bioinformatics analysis and chemical fragment analysis is a promising method for *in silico* “panning” or “screening” of proteins and pathways that EGCG may affect and thereby reduce risk of breast cancer. This approach may be suitable for analyzing the mechanism of action of other bioactive compounds. Our network analysis allowed us to identify several pathways by which EGCG may reduce cancer risk; these pathways are involved mainly in cell death and survival; DNA replication, recombination and repair; cell cycle; cellular assembly and organization; post-translational modification related to development; post-transcriptional modification; and protein synthesis. Our network analysis also allowed us to identify several specific proteins that EGCG may help regulate in breast cancer, including JUN, FADD, NFKB1, Bcl-2, GNAO1, and MMP14.

## Author contributions

Conceived and designed the experiments: XS. Analyzed the data: MZ, LC, QL. Wrote the paper: XS.

## Informed consent

Informed consent was obtained from all individual participants included in the study.

### Conflict of interest statement

The authors declare that the research was conducted in the absence of any commercial or financial relationships that could be construed as a potential conflict of interest.
