# Supplementary figures and images for: Bioinformatic Prediction of Possible Targets and Mechanisms of Action of the Green Tea Compound Epigallocatechin-3-Gallate Against Breast Cancer
Source: Front Mol Biosci. 2017 Jun 30;4:43. doi: 10.3389/fmolb.2017.00043 (PMC5492114; doi:10.3389/fmolb.2017.00043)

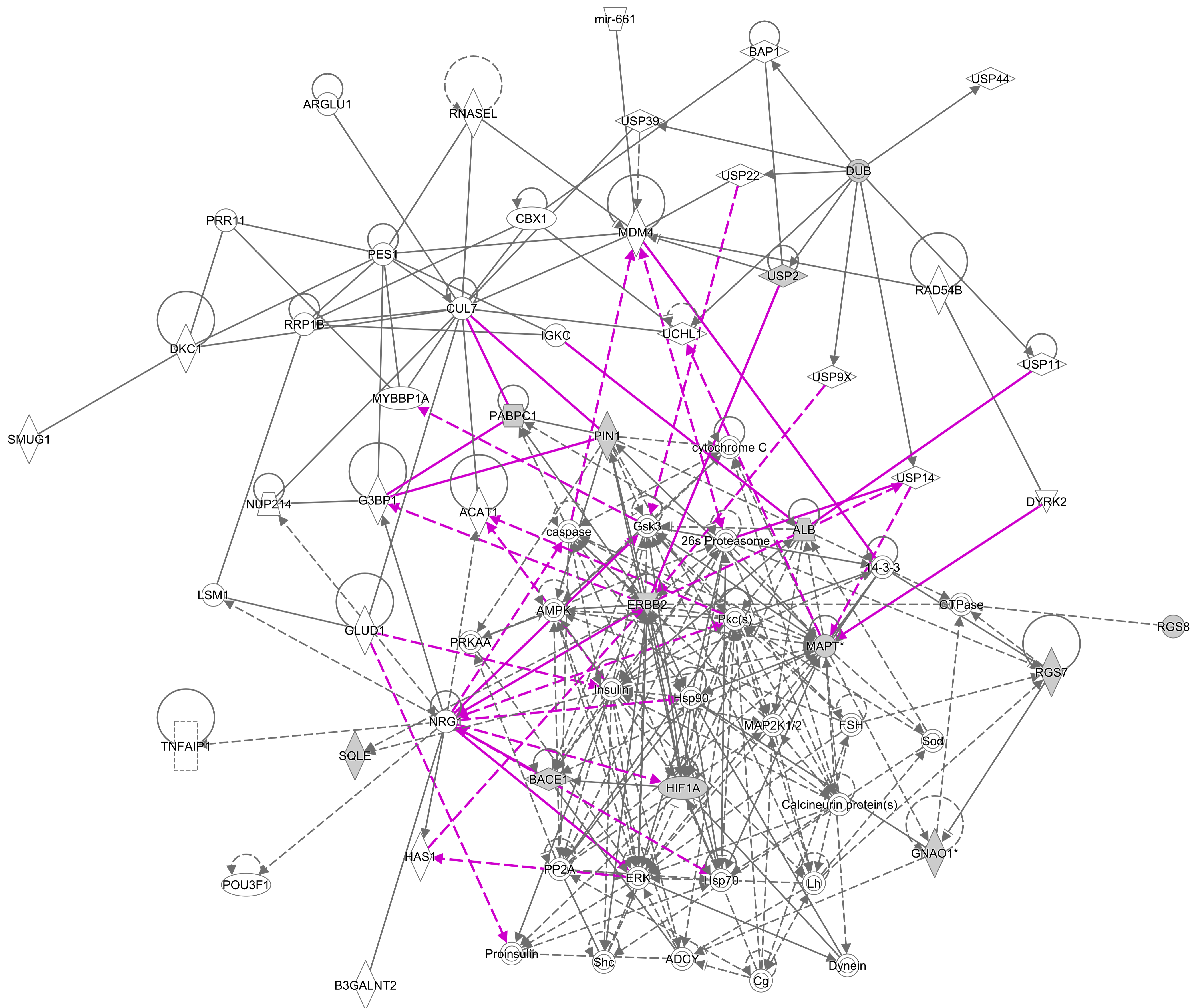

Supplement: Supplementary file 7 [file DataSheet2.zip › New folder/Figure 3_a_shared networks of EGCG and breast cancer.pdf]

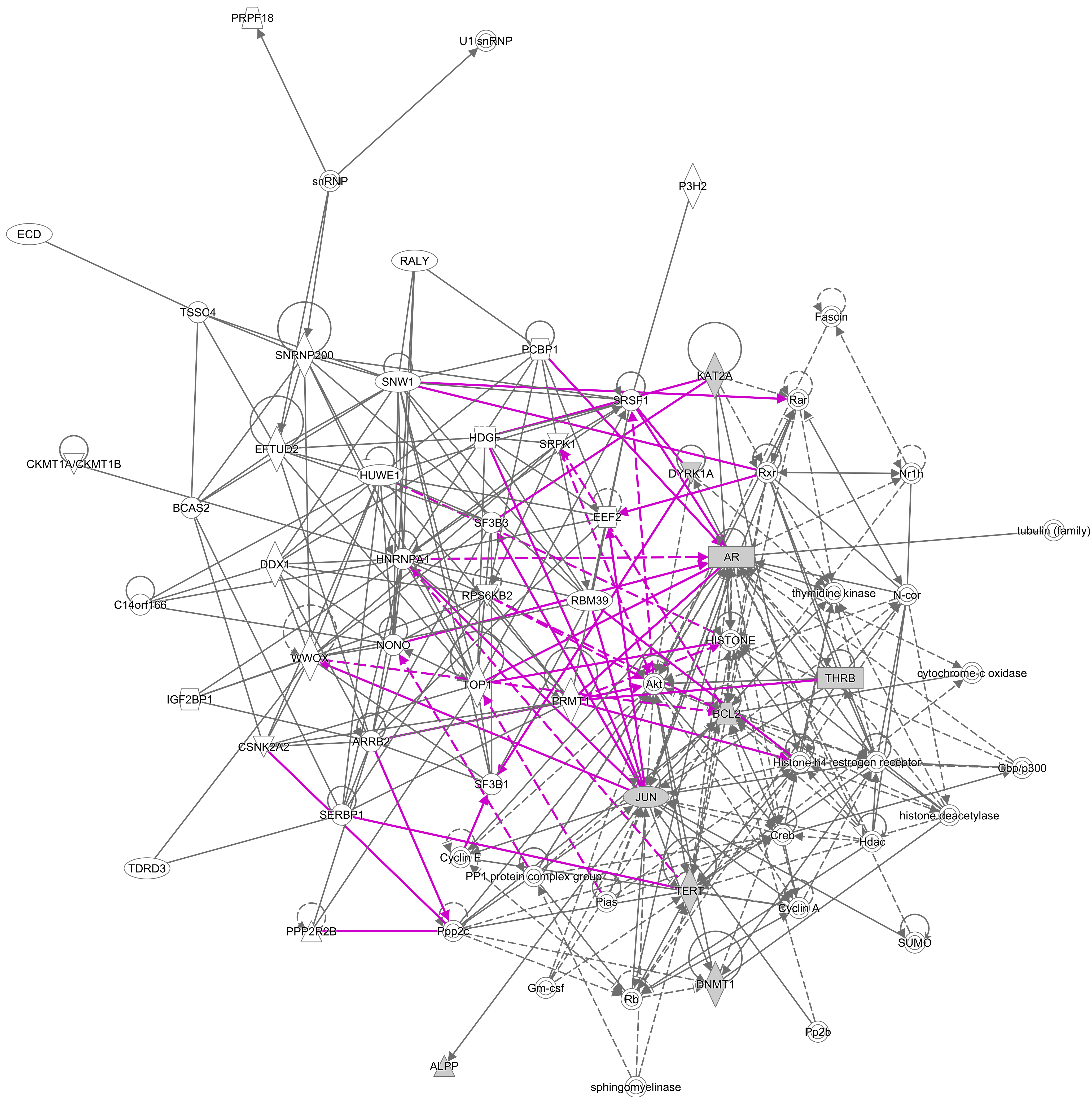

Supplement: Supplementary file 7 [file DataSheet2.zip › New folder/Figure 3_b_shared networks of EGCG and breast cancer.pdf]

Cytoplasm

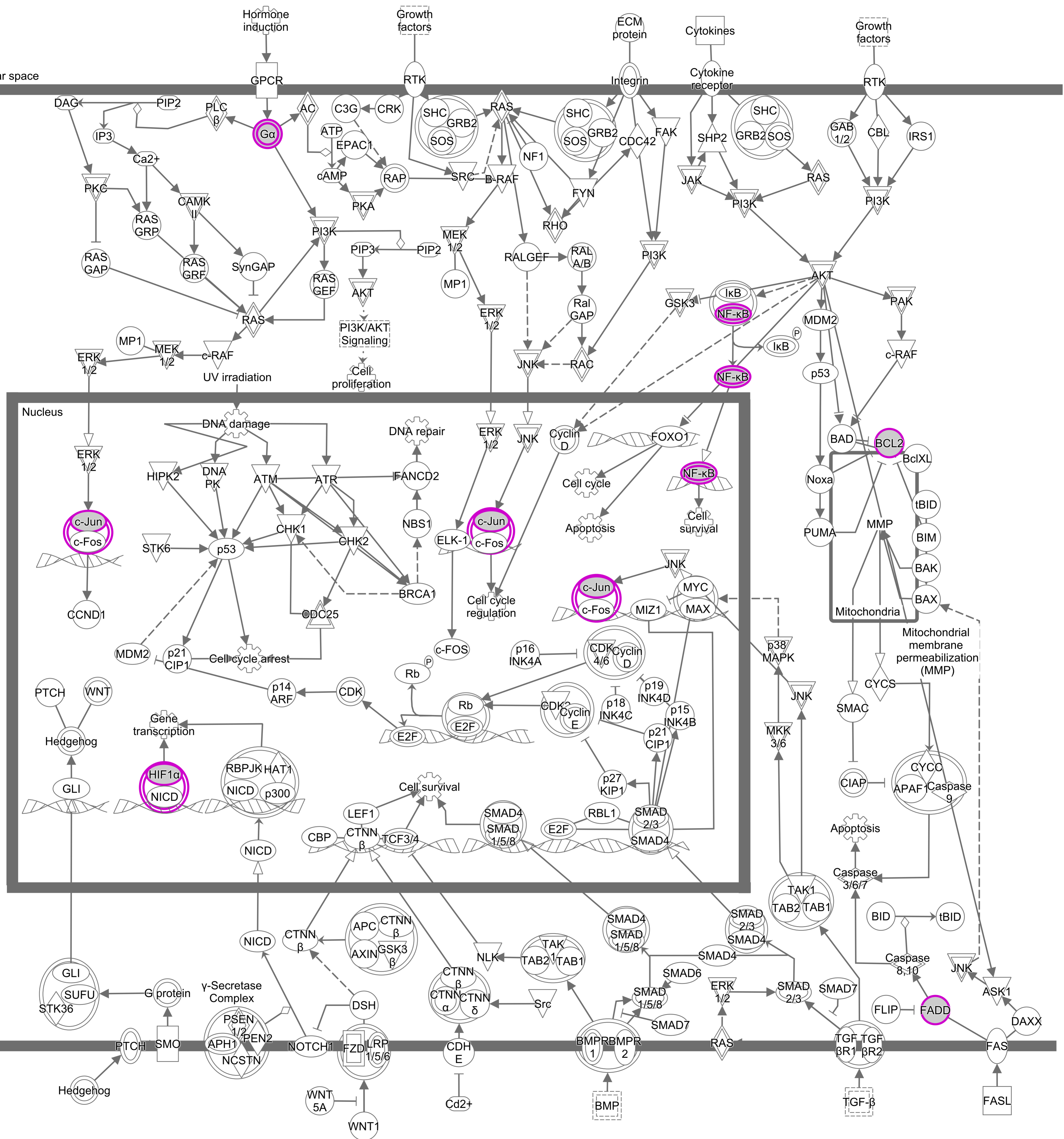

Supplement: Supplementary file 7 [file DataSheet2.zip › New folder/Figure 4_Molecular Mechanisms of Cancer.pdf]

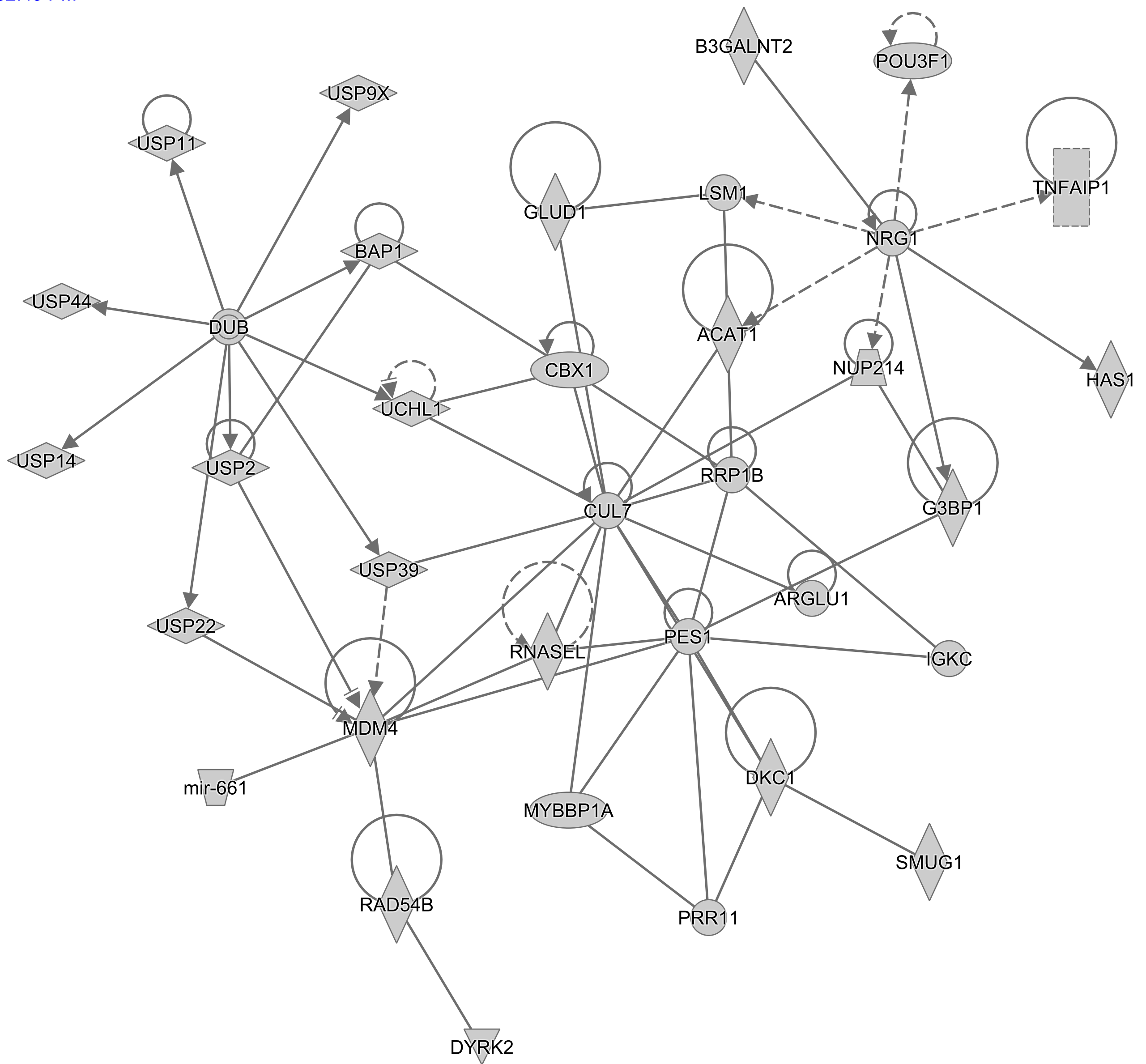

Supplement: Supplementary file 7 [file DataSheet2.zip › New folder/Figure S1_ breast cancer molecular network 1.pdf]

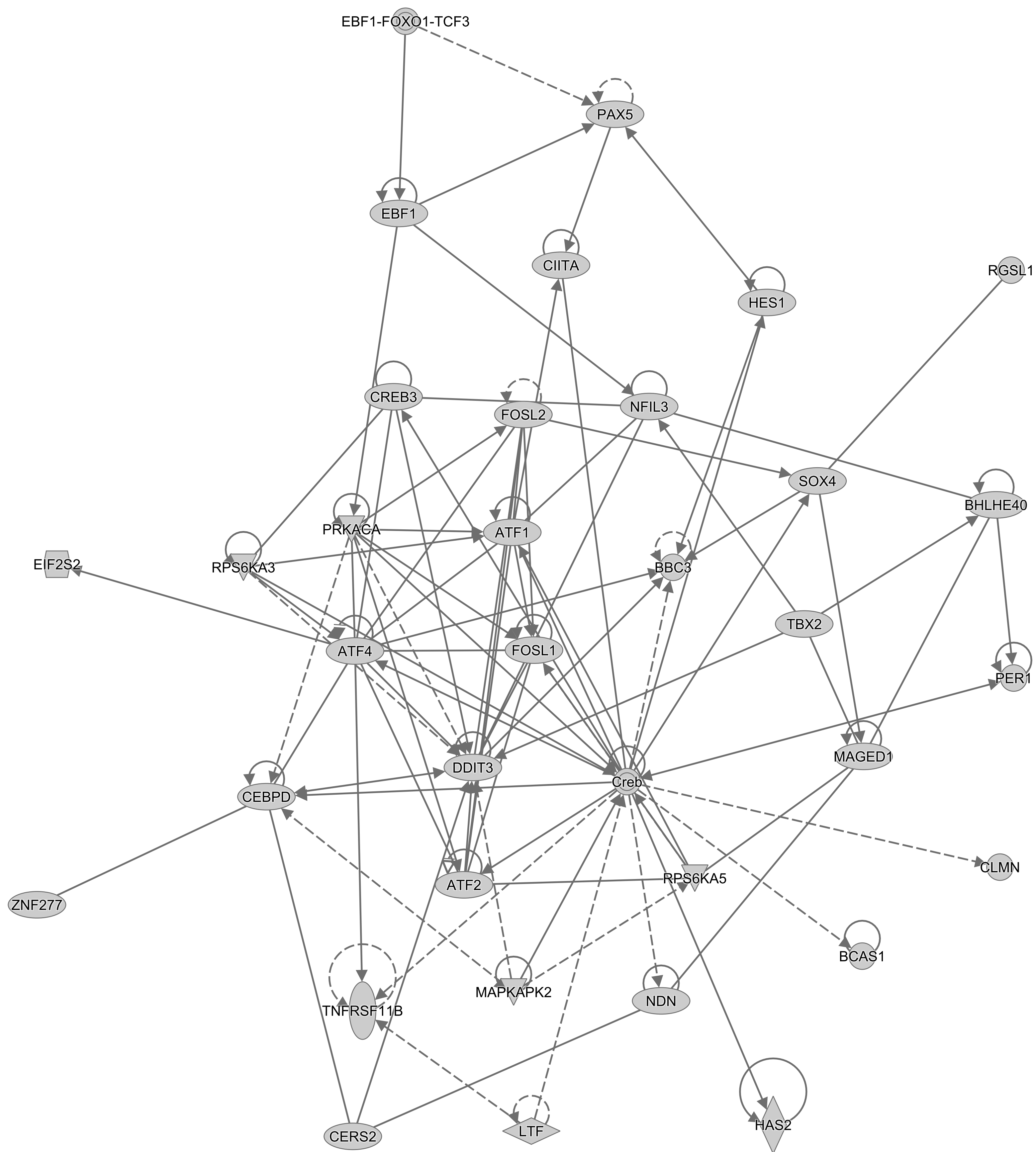

Supplement: Supplementary file 7 [file DataSheet2.zip › New folder/Figure S1_ breast cancer molecular network 3.pdf]

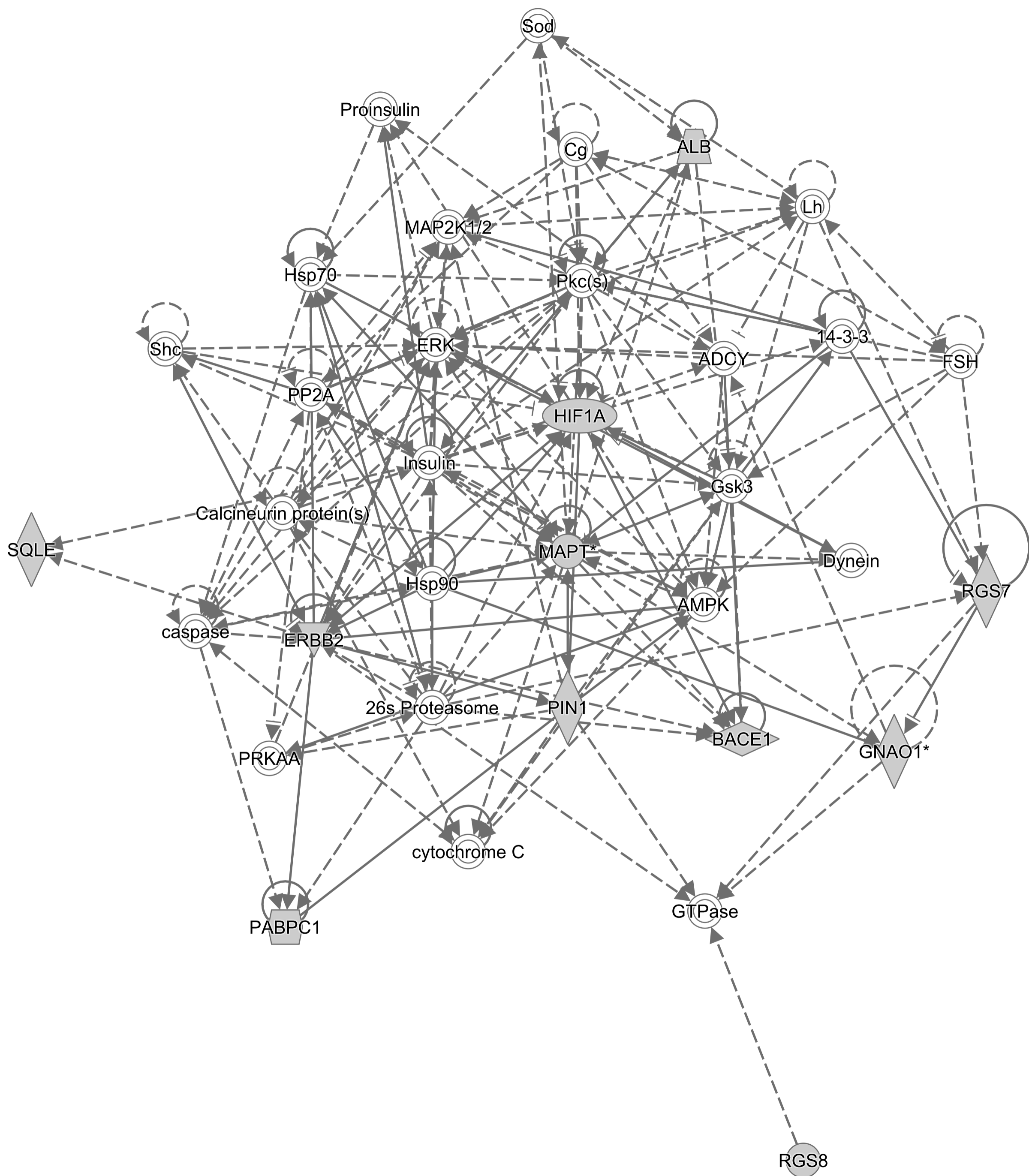

Supplement: Supplementary file 7 [file DataSheet2.zip › New folder/Figure S2_EGCG target proteins network 1.pdf]

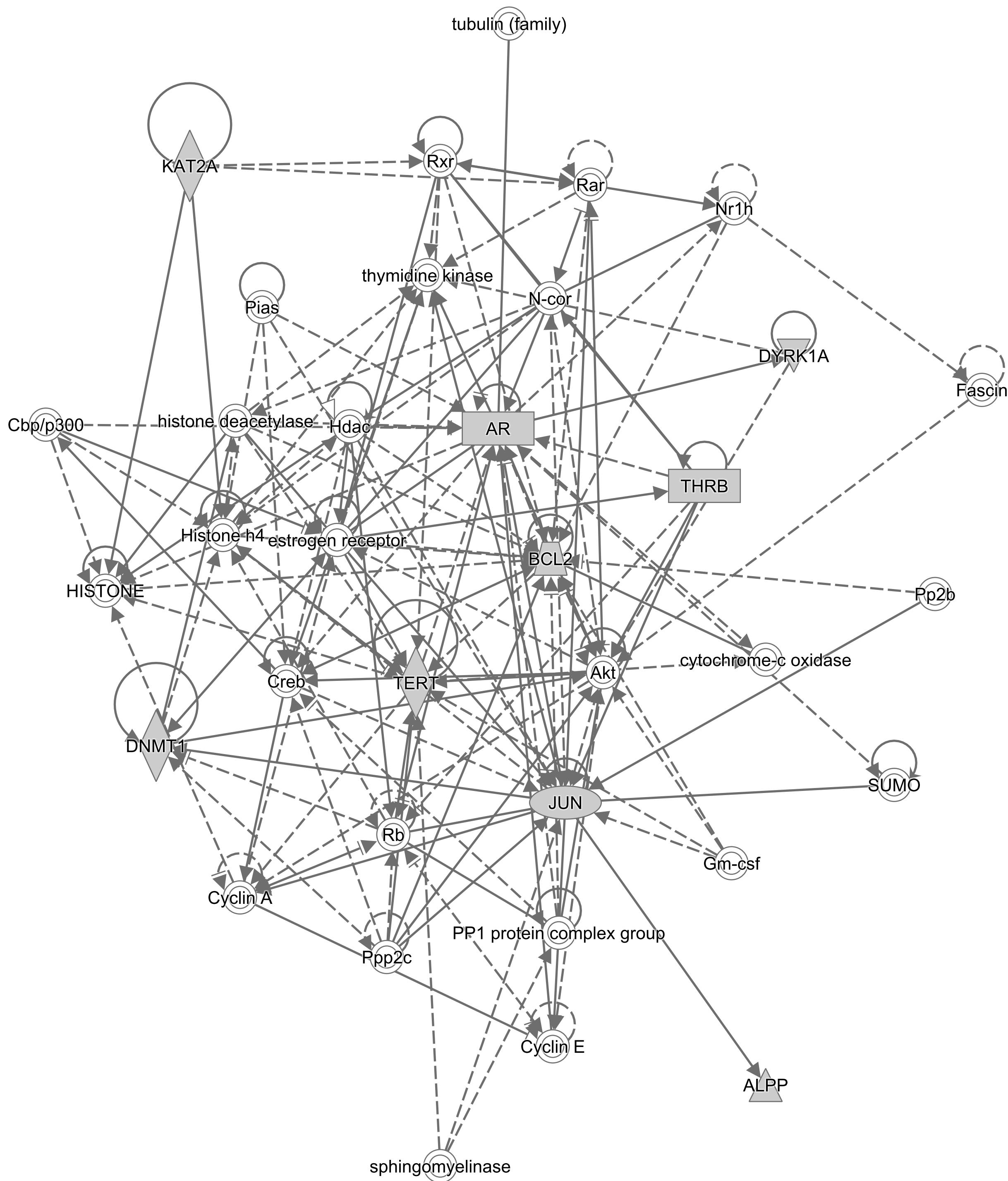

Supplement: Supplementary file 7 [file DataSheet2.zip › New folder/Figure S2_EGCG target proteins network 2.pdf]

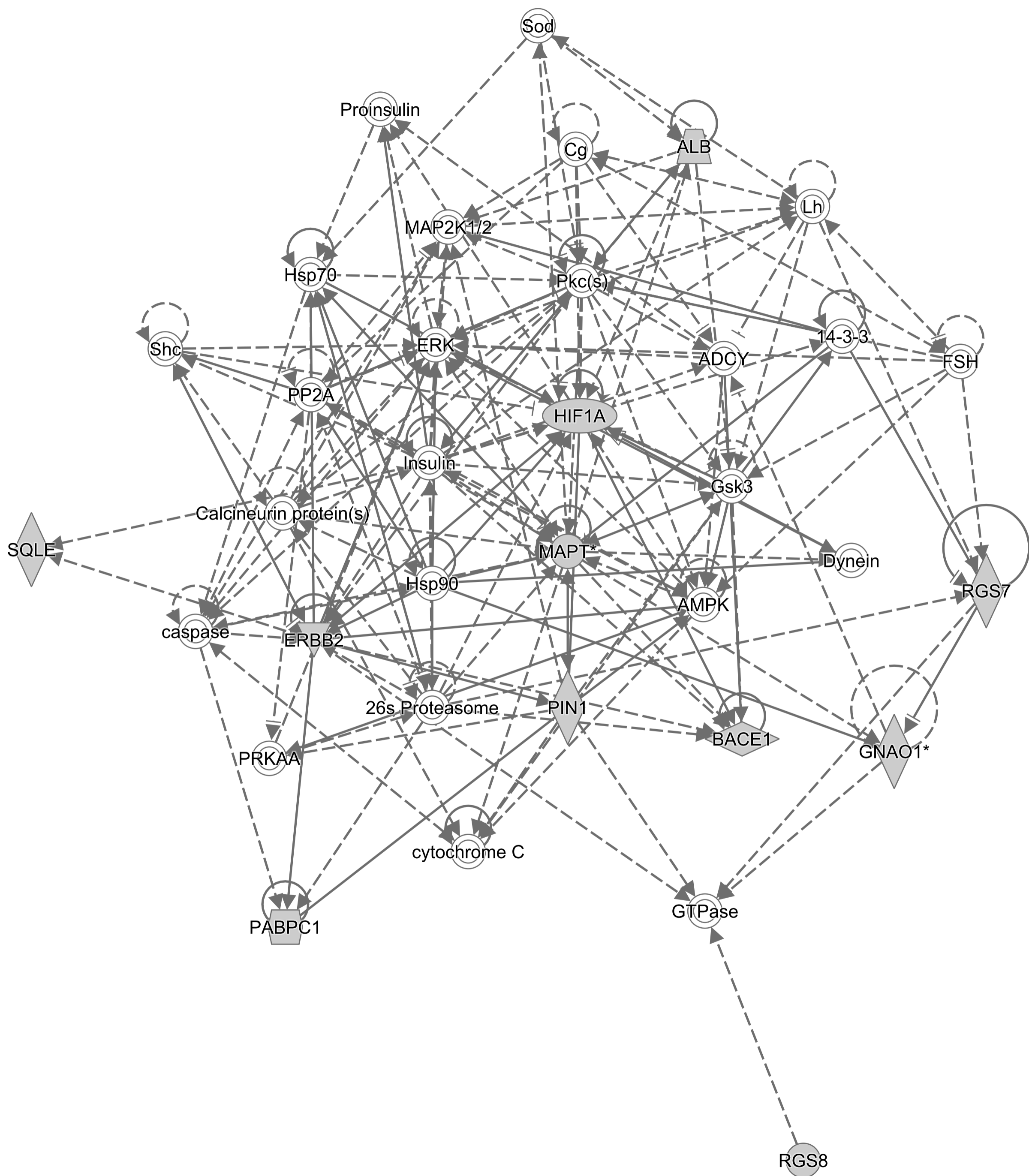

Supplement: Supplementary file 7 [file DataSheet2.zip › New folder/Figure S2_EGCG target proteins network 3.pdf]

Nucleus

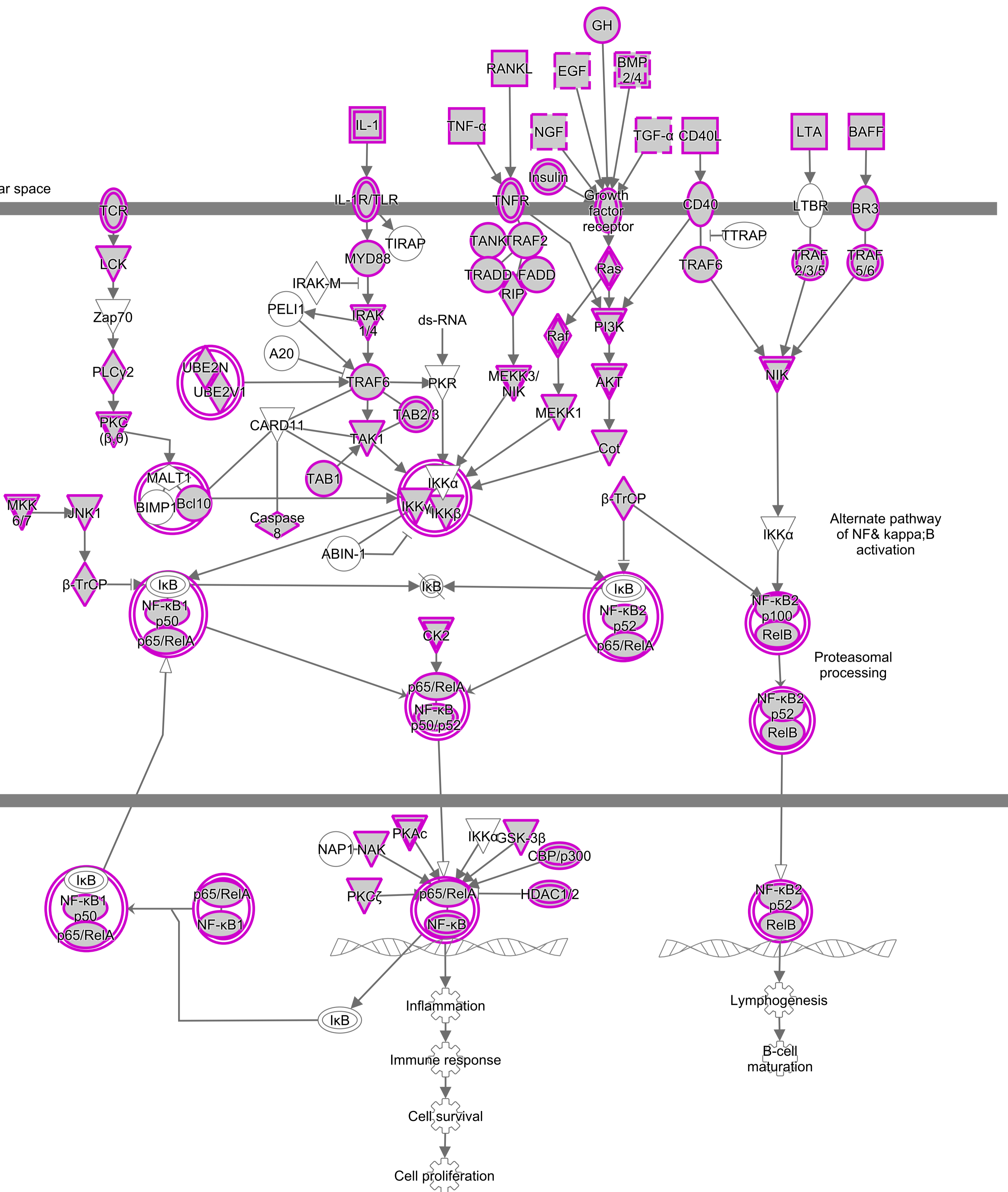

Supplement: Supplementary file 7 [file DataSheet2.zip › New folder/Figure S5_ common pathways of EGCG and breast cancer.pdf]
